# The Regulatory Network of METTL3 in the Nervous System: Diagnostic Biomarkers and Therapeutic Targets

**DOI:** 10.3390/biom13040664

**Published:** 2023-04-11

**Authors:** Xiaojuan Su, Yi Qu, Dezhi Mu

**Affiliations:** Department of Pediatrics, Key Laboratory of Birth Defects and Related Diseases of Women and Children, Ministry of Education, West China Second University Hospital, Sichuan University, Chengdu 610041, China

**Keywords:** METTL3, neurobiological events, neurological disorders, regulatory network, diagnostic biomarkers, therapeutic targets

## Abstract

Methyltransferase-like 3 (METTL3) is a typical component of N6-methyladenosine writers that exhibits methyltransferase activity and deposits methyl groups on RNA. Currently, accumulating studies have demonstrated the involvement of METTL3 in the regulation of neuro-physiological and pathological events. However, no reviews have comprehensively summarized and analyzed the roles and mechanisms of METTL3 in these events. Herein, we are focused on reviewing the roles of METTL3 in regulating normal neurophysiological (Neurogenesis, Synaptic Plasticity and Glial Plasticity, Neurodevelopment, Learning and Memory,) and neuropathological (Autism Spectrum Disorder, Major Depressive Disorder, Neurodegenerative disorders, Brain Tumors, Brain Injuries, and Other Brain Disorders) events. Our review found that although the down-regulated levels of METTL3 function through different roles and mechanisms in the nervous system, it primarily inactivates neuro-physiological events and triggers or worsens neuropathological events. In addition, our review suggests that METTL3 could be used as a diagnostic biomarker and therapeutic target in the nervous system. Collectively, our review has provided an up-to-date research outline of METTL3 in the nervous system. In addition, the regulatory network for METTL3 in the nervous system has been mapped, which could provide directions for future research, biomarkers for clinical diagnosis, and targets for disease treatment. Furthermore, this review has provided a comprehensive view, which could improve our understanding of METTL3 functions in the nervous system.

## 1. Introduction

**Figure 1:**
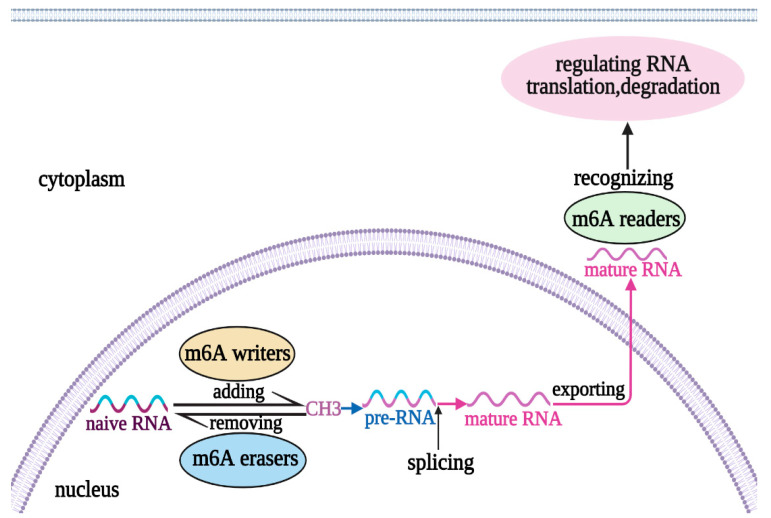
Mechanism of RNA m6A Modification. The process of m6A modification on RNA consists of adding and removing a methyl group by writers and erasers to form pre-RNA and then splicing to form the mature RNA, respectively. Finally, the mature RNA was nuclear-exported, and recognized by readers to regulate RNA translation and degradation. m6A, N6-methyladenosine.CH3, methyl group.

N6-methyladenosine (m6A) is an epigenetic modification of RNAs [1], which involves adding (via writers) or removing (via erasers) a methyl group at the N6-position of adenosine [2]. Writers include methylases such as METTL3, METTL14, and WT1-associated protein (WTAP), and erasers include demethylases such as fat mass and obesity-associated protein (FTO) and AlkB homolog 5(ALKBH5) [3]. The modification is recognized by m6A reader proteins (such as YTHDF domain-containing proteins) [3] (Figure 1). Therefore, the m6A modification is a carefully controlled and regulated process. Specifically, the normal performance of m6A modification is critical for the regulation of physiological processes [4]; however, dysregulation of this modification contributes to multiple pathological processes [4]. In summary, it is easy to recognize the significance of m6A components in the regulation of physiological and pathological processes in the body.

METTL3 is an important component of m6A writers, with typical structures and functions, that mainly acts as a methylase in m6A regulation [3]. Previous studies have indicated that METTL3 participates as a dynamic epigenetic regulator involved in the regulation of neurophysiological and -pathological events [5]. Physiological events include nerve cell biological functions, neurogenesis, learning and memory, and neurodevelopment. Pathological events include brain tumors (glioblastoma, GBM), neurodegenerative diseases (Alzheimer’s disease, AD), brain injuries (e.g., transient focal ischemia and traumatic brain injury (TBI)), and other brain disorders (e.g., cerebral arteriovenous malformations, typical neuropathic pain, and postoperative cognitive dysfunction). However, reviews that comprehensively summarize and analyze the roles and mechanisms of METTL3 in nervous system regulation are lacking. 

Herein, by reviewing METTL3 studies and further analyzing the biological roles and molecular mechanisms of METTL3 in regulating gene expression in the nervous system, we aim to (1) comprehensively highlight the multifaceted roles and mechanisms of METTL3 in the regulation of neurophysiological and neuropathological processes, and (2) map a regulation network for METTL3, which provides directions for future research, biomarkers for clinical diagnosis, and targets for disease treatment. Collectively, this review has provided a comprehensive view, which could improve our understanding of METTL3 functions in the nervous system. 

## 2. Structure and Function of METTL3

METTL3, a 70 kDa protein also named MT-A70, was first identified and isolated from mammalian cell nuclear extracts in 1997 [6]. It belongs to the conserved family of methyltransferases, possessing a methyltransferase domain [MTD] that catalyzes methyl transfer to adenosine [6]. The N-terminus of METTL3 contains two Cys-Cys-Cys-His (CCCH)-type zinc finger (ZnF) motifs common in RNA-binding proteins [7]. The helical structure at the N-terminus of METTL3, also known as the leader helix, is necessary for METTL3 and WTAP interaction. The binding site of METTL3 is located within the first 150 amino acids of WTAP, and the main interaction of METTL3 with METTL14 is mediated by the MTD [7]. Typically, METTL3 and METTL14 first form a heterodimeric enzyme complex (METTL3/METTL14) in the nucleus; the METTL3/METTL14 complex is restricted to nuclear speckles and then interacts with WTAP to form the m6A methyltransferase complex (METTL3/METTL14/WTAP) that is also called the m6A writer [8,9]. 

METTL3/METTL14 usually functions by adding the methyl group, while WTAP potentially regulates the recruitment of this complex to mRNA targets [10]. METTL3 is the key component of the m6A writer that has critical functions in the activity of the METTL3/METTL14/WTAP complex, including catalysis, binding, and translational regulation [10]. One study indicated that the RNA-binding capacity of METTL3 is highly reduced in the absence of WTAP and that it primarily acts as a methylase to exert its core catalytic functions during m6A modification, while METTL14 serves as the RNA-binding platform [11]. Therefore, the catalytic activity of the complex is solely conferred by METTL3, which favorably modifies substrate RNAs containing the GGACU consensus sequence. Another study indicated that METTL3 could recognize 3′-UTR m6A sites on target mRNAs in addition to methyltransferase activity and then promote protein translation of the transcript by facilitating translation loop formation via interaction with the eukaryotic translation initiation factor 3 (eIF3h) subunit [11]. Collectively, these findings suggest that the typical structure of METTL3 determines its specific functions. Moreover, these functions have been implicated in the physiological and pathological processes of the nervous system.

## 3. METTL3 in Neurophysiological Events

METTL3 influences the behavior of RNA in nerve cells, which in turn regulates cell biological functions. Based on the roles of METTL3 in nerve cells, it further regulates many neurobiological events such as neurogenesis, learning and memory, and neurodevelopment.

### 3.1. METTL3 with Neurogenesis

Neurogenesis is the formation of new neurons and glial cells from neural stem cells [12]. Neural stem cells (NSCs) can differentiate into neural progenitor cells and glial progenitor cells; the latter produce glial cells such as astrocytes, oligodendrocytes, and microglia. The regulated process of neurogenesis produces an incredible diversity of neurons to carry out the complex functions of the brain [12]. Therefore, neurogenesis is accompanied by the regulation of nerve cell biological functions. Currently, several studies have indicated the multifaceted roles and mechanisms of METTL3 in the regulation of nerve cell biological processes [13]. For example, Choi et al. [14] demonstrated the role of METTL3 in cell reprogramming, reporting that METTL3 knockdown decreased the efficiency of direct lineage reprogramming, whereas METTL3 overexpression increased the efficiency of the induced neuronal cell (iN) generation [14]. The transcription factor B-cell translocation gene 2(Btg2) is a functional target of METTL3 for efficient iN generation [14]. Together, these findings indicate the importance of METTL3 modification in remodeling cell fate transition into iNs. In addition, METTL3 is also involved in the regulation of nerve cell proliferation. Chen et al. reported that the depletion of METTL3 significantly reduced m6A levels in adult NSCs, inhibiting their proliferation [15]. Meanwhile, another study in glioma stem-like cells (GSCs) also indicated that METTL3 expression is downregulated [16]. Further investigation identified SOX2 as its target in maintaining GSC stability. Moreover, silencing METTL3 promoted the proliferation of GSCs, which was achieved by the alternative splicing of isoform switches and modulating the nonsense-mediated mRNA decay of splicing factors in GBM [16,17]. Barring the above cell biological functions, cell differentiation plays a critical role during a cell’s life and has also been reported to be regulated by METTL3. For example, Yoon et al. found that METTL3 knockdown led to the prolongation of the cell cycle and maintenance of radial glial cells, which hindered cell differentiation [18]. Besides, Visvanathan et al. reported that METTL3 expression is downregulated in GSCs during differentiation by targeting SOX2 [17]. Collectively, these studies demonstrated that the downregulated expression of METTL3 actively participated in inhibiting the proliferation, reprogramming, and differentiation of nerve cells via different pathways, hinting that METTL3 might regulate neurogenesis as well.

**Figure 2:**
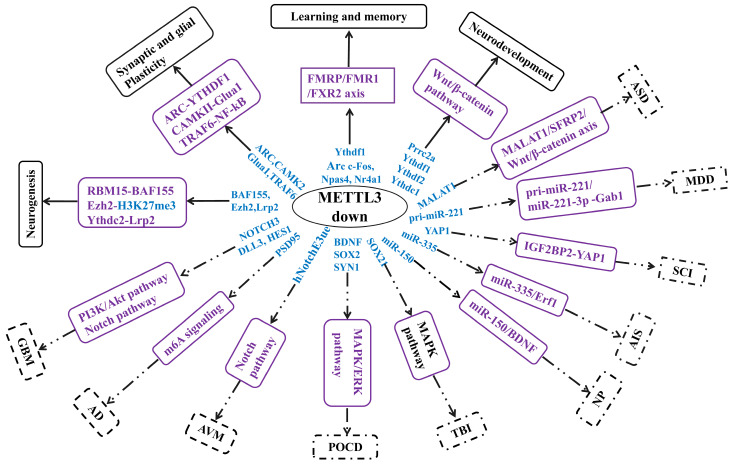
Illustration of the regulatory network of METTL3 in the nervous system. The targets and signaling pathways of METTL3 in neurophysiological and neuropathological events are summarized. We found that low expression of METTL3 exhibits different effects, which exert beneficial roles to promote neurological events such as promoting neurogenesis, while possibly exacerbating neuropathological events, such as worsening GBM. Blue wording, targets. Purple wording, signaling pathways. Solid box, neurophysiological events. Dotted box, neuropathological events. METTL3, methyltransferase-like3; METTL3 downregulation facilitated glioma (GBM) occurrence by altering the PI3K/Akt pathway, and promoted glioma development via activating the Notch pathway that targeted NOTCH3, DLL3, and HES1; AD, METTL3 downregulation worsens Aβ-induced synaptic damage in Alzheimer’s disease (AD) via m6A signaling that is mainly targeted to upregulation of PSD95; downregulation of METTL3 significantly affected angiogenesis of human endothelial cells in arteriovenous malformation (AVM) by reducing the level of hNotch E3ue and activating the Notch signaling pathway; METTL3 downregulation participates in sevoflurane-induced postoperative cognitive dysfunction (POCD) via suppressing the MAPK/ERK pathway that targeted BDNF, SOX2, and SYN1; METTL3 downregulation triggered traumatic brain injury (TBI) development via the MAPK signaling pathway that downregulated SOX21 expression; NP, METTL3 downregulation triggers neuropathic pain (NP) occurrence via the miR-150/BDNF pathway; METTL3 downregulation triggers acute ischemic stroke (AIS) occurrence via the miR-335/Erf1 axis. NOTCH3, Notch homolog 3; DLL3, Delta-like 3; PSD95, postsynaptic density protein 95; hNotch E3ue, heterodimeric Notch E3 ubiquitin ligase; BDNF, neurotrophic factor; SYN1, synapsin 1; IEGs, immediate early genes; Ezh2, enhancer of Zeste homolog 2.

Accumulating evidence suggests the involvement of METTL3 in neurogenesis regulation. One study indicated that the ablation of RNA-binding motif protein 15 (RBM15, a subunit of the m6A methyltransferase complex) expression in cultured neuronal cells and the developing cortex augmented chromatin remodeling factor Brg1/Brm-associated factor 155 (BAF155) expression, which is one of the integral subunits of the SWI/SNF-like complex that uses ATP-derived energy to regulate nucleosome occupancy and chromatin architecture [19]. Conversely, RBM15 overexpression decreased BAF155 expression. Mechanistically, transcriptional profiling found that BAF155 mRNA degradation by RBM15 depends on METTL3 activity, which disrupts the ability of BAF155 to produce the apical radial glial progenitors that are a hallmark of the genesis of basal radial glial progenitor cells [19]. This study detailed the key roles of METTL3 in regulating glial progenitor genesis, which is a significant step in neurogenesis. Another study found that the depletion of METTL3 inhibited neuronal development, disrupted the differentiation of adult NSCs more toward glial lineage, and affected the morphological maturation of newborn neurons in the adult brain [15]. Further mechanistic investigation revealed that METTL3 knockdown decreased both histone methyltransferase Ezh2 protein expression and consequent H3K27me3 levels [15]. Furthermore, Ezh2 overexpression could rescue the neurogenesis and neuronal development defects induced by METTL3 depletion. These findings indicate that METTL3-mediated m6A modification plays an important role in regulating neurogenesis and neuronal development by targeting Ezh2 [15]. In addition, in an adult hippocampal neurogenesis study, it was shown that the knock-down of METTL3 in NSCs dramatically reduced proliferation and neuronal genesis, while enhancing glial differentiation [20]. Mechanically, METTL3 enhances the stability and translation efficiency of Lrp2 mRNA by relying on the reader protein Ythdc2, which in turn promotes neurogenesis [20]. Consistently, depletion of METTL3 in mice also showed reduced hippocampal neurogenesis, decreased spatial memory, and depression-like behavior [20]. Overexpression of either METTL3 or Lrp2 in the hippocampus of depressed mice may rescue these behavioral deficits [20]. Collectively, these findings demonstrate that METTL3 modulates adult hippocampal neurogenesis by targeting the Ythdc2-Lrp2 axis (Figure 2).

### 3.2. METTL3 with Synaptic Plasticity and Glial Plasticity

Consistently, recent studies have also hinted at the role and mechanisms of METTL3 in regulating synaptic and glial plasticity. For example, the study by Mareen Engel et al. group reported that METTL3 was upregulated in the amygdala (AMY) after acute stress [21], which rescued the synaptic plasticity impairment as time goes on via upregulating the m6A methylation of its target gene Ntrk1, Creb1, and Nr3c1 in the hippocampus, suggesting that METTL3 upregulation in AMY is closely related to maintaining synaptic plasticity [21]. In addition, a study on the freezing and anoxia of the wood frog brain indicated that METTL3 was significantly upregulated, coupled with a significant increase in stress granule (SG) markers TIAR and TIA-1, indicating the beneficial role of METTL3 in balancing synaptic plasticity to confront the low-oxygen stress [22]. Moreover, loss of Mettl3 decreased the levels of nuclear Ythdc1, which in turn leads to stress resilience. Overall, these data suggest that METTL3-mediated m6A modification on YTHDC1 in Drosophila dampens the brain’s biological response to stress [23]. Activity-regulated cytoskeleton-associated protein (ARC) plays an important role in the synaptic plasticity of memory consolidation [24]. The study from analyzing the brain of AD patients and Aβ-induced cell models indicated that METTL3 was downregulated to inhibit ARC expression via YTHDF1-dependent m6A modification, which then impaired the synaptic plasticity to influence memory capacity, while overexpression of METTL3 rescued ARC expression after Aβ treatment [24]. These findings demonstrate the role of low levels of METTL3 in triggering or exacerbating synaptic plasticity via the ARC-YTHDF1 axis. Moreover, the study from the Ricardo Castro-Hernándeza group reported that neurons with METTL3 knockdown significantly impaired the synaptic translation of calcium/calmodulin-dependent protein kinase 2 (CAMK2) and AMPA-selective glutamate receptor 1 (Glua1) mRNAs [25]. Consistently, the knockdown of METTL3 was also associated with decreased neuronal activity [25]. Collectively, these findings provide evidence for the idea that METTL3-mediated down-regulation of the m6A-modification mechanism contributes to synaptic function impairment via impairing the synaptic proteins CAMKII and Glua1 synthesis.

Consistent with the regulation of synaptic plasticity, there have been several studies hinting at the role and mechanism of METTL3 in regulating glial plasticity. For example, Wen et al. reported that METTL3 expression was increased in LPS-induced microglial inflammation, which subsequently upregulated TRAF6 m6A modification to activate the NF-κB pathway [26]. Consistently, inhibiting NF-κB attenuated METTL3-mediated microglial activation [26]. Collectively, these findings suggested that the upregulated level of METTL3 triggered the pro-inflammation activity of microglia by activating the TRAF6-NF-κB pathway. Furthermore, in a retina-specific METTL3 conditional knockout mouse model [27], Xin et al. reported that the deletion of METTL3 promoted the degradation of retinal progenitor cell (RPC) transcripts and subsequently promoted the transition of RPCs into glial cells, indicating that a low level of METTL3 triggers glial cell formation, which is usually related to neurological disease pathogenesis [27].

### 3.3. METTL3 with Neurodevelopment

Emerging studies have indicated that METTL3 is temporally and spatially regulated during neurodevelopment and aging [28]. METTL3 mRNA expression shows a distinct tissue-specific methylation profile that is associated with tissue-specific developmental processes. For example, in cortical-specific conditional FTO and METTL3 double knockout mice, Du et al. showed that the resultant severe brain defects were caused by METTL3 and not FTO deletion [29]. Moreover, METTL3 deletion elevated translation of Prrc2a, Ythdf1, Ythdf2, and Ythdc1 [29]. In summary, this study uncovered a profound role of METTL3 in regulating the translation of major mRNAs that control proper cortical development by influencing “reader” proteins. In addition, depletion of m6A by METTL3 knockout also leads to a prolonged cell cycle and maintenance of radial glial cells, which hinders cortical neurogenesis, suggesting the role of METTL3 in maintaining normal neurogenesis [18] (Figure 2).

The hippocampus and AMY are two regions that respond to several behaviors, including memory. The study showed that METTL3 depletion alters not only the steady-state transcriptome in adult hippocampal neurons, but also the transcriptomic response to fear conditioning stress, including regulation of several genes involved in neuronal circuit function and pointing out a function of m6A/m in regulating neuronal circuits [21]. Moreover, it has also been revealed that METTL3 knockout significantly hypermethylated genes in the AMY, which regulate behavioral and hormonal stress responses, fear, and anxiety [21]. Collectively, these data suggest that METTL3 is critical for maintaining the normal development of the hippocampus and AMY.

Furthermore, METTL3 is actively involved in the regulation of the developing mouse cerebellum. Wang et al. inactivated METTL3 specifically in the developing mouse brain and reported severe developmental defects in the cerebellum [30]. Further analysis indicated that METTL3-mediated m6A participates in cerebellar development by controlling the mRNA stability of genes related to cerebellar development or apoptosis and by regulating the alternative splicing of pre-mRNAs of synapse-associated genes [30]. Another study showed the spatiotemporal-specific expression of METTL3 in the mouse cerebellum, and ectopic expression of METTL3 mediated by lentiviral transduction led to disorganized structures of both Purkinje and glial cells [31] (Figure 2).

Apart from its roles in cortical and cerebellar development, METTL3 can regulate neural tube development (NTD). Zhang et al. reported that METTL3 was enriched in HT-22 cells, and its depletion reduced cell proliferation and increased apoptosis by suppressing the Wnt/β-catenin signaling pathway [32]. This research revealed the important role of METTL3 in regulating NTD. Another study indicated that the knockdown of METTL3 caused anteriorization of neurulas and tailbud embryos along with the loss of the neural crest and neuronal cells in the Xenopus [33]. The mechanistic analysis determined that canonical Wnt signaling was inhibited in METTL3 morphants, which might explain the neural patterning defects of the morphants [33]. This study addressed the multiple roles of METTL3 during Xenopus neurulation in anteroposterior neural patterning, neural crest specification, and neuronal cell differentiation, which further supports the hypothesis that METTL3 plays significant roles during NTD (Figure 2).

Taken together, these findings suggest that the down-regulated expression of METTL3 plays a negative role in spatiotemporal fashions in both early and late brain development.

### 3.4. METTL3 with Learning and Memory

Learning and memory are dominant functions of the brain. Hippocampal neurogenesis is critical in forming new memories [34]. A study has shown that dysregulated neurogenesis in the hippocampus severely impairs learning and memory tasks that are dependent on the hippocampus [35].

As METTL3 broadly participates in the process of neurogenesis, the neurogenesis-dependent functions of learning and memory are also greatly altered by METTL3. Shi et al. reported that mice with hippocampus-specific knockdown of METTL3 exhibited learning and memory defects, as well as impaired hippocampal synaptic transmission and long-term potentiation, whereas overexpression of METTL3 rescued the neurobehavioral abilities and synaptic defects in mice by targeting YTH N6-methyladenosine RNA binding protein 1(YTHDF1) [36]. This study emphasized the significant roles of METTL3 in maintaining normal hippocampal function, suggesting that the abundant METTL3 in the wild-type mouse hippocampus is positively correlated with learning efficacy, and overexpression of METTL3 significantly enhances long-term memory consolidation [36]. Another study reported that METTL3 depletion in the mouse hippocampus could reduce memory consolidation ability by translational regulation of immediate early genes, including Arc, c-Fos, Egr1, Npas4, and Nr4a1, while unimpaired learning outcomes could be achieved if the function of METTL3 was restored [37]. These findings uncovered a direct role of METTL3 modification in regulating long-term memory formation. Apart from the role of METTL3 in memory formation, it was also found to be associated with the regulation of memory types, such as fear memory [21]. METTL3 deletion in adult neurons altered the FMRP/FMR1 and FXR2 transcriptome response to fear and synaptic plasticity, and finally increased fear memory [21]. Moreover, the study reported that neither deletion of METTL3 nor FTO in mice showed altered anxiety-like behavior or locomotion, but we observed significant changes in spontaneous digging behavior [21] (Figure 2).

## 4. METTL3 in Neuropathological Events

Beyond regulating normal physiological processes of the nervous system, METTL3 is also involved in the regulation of various pathological events that occur in the nervous system, including brain tumors, neurodegenerative diseases, and brain injuries. 

### 4.1. METTL3 with Autism Spectrum Disorder 

Autism spectrum disorder (ASD) is one of the most common neurodevelopmental disorders, and hippocampal neuron apoptosis is greatly associated with the occurrence of ASD [38]. Ming et al. reported that METTL3 was significantly downregulated in the hippocampal tissue of a mouse model of ASD, which facilitated the m6A modification of MALAT1 and subsequent stabilization of MALAT1 expression [39]. Next, MALAT1 decreased the expression of SFRP2 by recruiting DNMT1, DNMT3A, and DNMT3B to the SFRP promoter region, and SFRP2 finally promoted the activation of Wnt/β-catenin signaling. Mechanically, downregulation of METTL3 triggered hippocampal neuron apoptosis by regulating the MALAT1/SFRP2/Wnt/β-catenin axis to trigger ASD occurrence and development [39] (Figure 2).

### 4.2. METTL3 with Major Depressive Disorder

Since METTL3 is essential for maintaining the normal development of the hippocampus and AMY, which is primarily responsible for behavioral regulation, its abnormal regulation also results in neurodevelopmental disorders [40]. For example, major depressive disorder (MDD) is a mental health problem that is exhibited as an enormous impairment in mood, cognition, and memory and systemic inflammation, and it further harms hippocampal neurogenesis [41]. Niu et al. reported that METTL3 was highly expressed in chronic unpredictable mild stress (CUMS)-induced MDD rats [42]. Further mechanic study found that METTL3-mediated m6A modification on pri-miR-221 promoted its processing and maturation that subsequently upregulated miR-221-3p expression to inhibit GRB2-associated binding protein 1 (Gab1) expression, which worsened the cognitive deficits in MDD rats [42] (Figure 2). 

### 4.3. METTL3 with Neurodegenerative Disorders

Neurodegenerative diseases are primarily characterized by the loss of neurons, causing people to have problems moving, thinking, feeling, or behaving [43]. AD is a progressive brain disease characterized by changes in the brain, including amyloid plaques formed by β-amyloid protein deposition, neurofibrillary tangles formed by the hyper-phosphorylation of tau proteins, and neuronal loss caused by glial cell proliferation, resulting in the loss of neurons and their connections exhibited as memory loss and progressive cognitive impairment in patients [44]. In an APP/PS1 transgenic mouse model of AD, Han et al. reported that m6A methylation was elevated in the cortex and hippocampus [45]. Further investigation revealed that the expression of METTL3 was elevated and that of FTO was decreased in AD mice. Gene Ontology and Kyoto Encyclopedia of Genes and Genomes analyses indicated that mRNAs modified by m6A were primarily associated with synaptic or neuronal development and growth [45]. Additionally, in postmortem brain tissue samples from humans with AD, researchers found that METTL3 and RBM15B were downregulated and upregulated, respectively, in the hippocampus [46]. Accumulation of METTL3 was found in the insoluble fractions, which was positively correlated with the levels of insoluble Tau protein in the postmortem samples [46]. These findings indicate that the aberrant expression and distribution of METTL3 in the hippocampus of the AD brain may represent an epi transcriptomic mechanism for the pathogenesis of AD [46]. Similarly, Yin et al. reported that METTL3 downregulation in monocyte-derived macrophages improved cognitive function in an amyloid beta (Aβ)-induced AD mouse model [47]. Mechanistically, METTL3 downregulation reduced m6A modification in DNA methyltransferase 3A (DNMT3A) mRNAs and consistently inhibited YTHDF1-mediated DNMT3A translation. In addition, DNMT3A bound to the promoter region of alpha-tubulin acetyltransferase 1 (ATAT1) and maintained its expression [47]. Therefore, METTL3 depletion resulted in the downregulation of ATAT1, reduced acetylation of α-tubulin, and subsequently enhanced migration of monocyte-derived macrophages and Aβ clearance [47]. Collectively, these findings provide a novel METTL3/YTHDF1-DNMT3A-ATAT1 pathway for alleviating AD symptoms, suggesting that MEETL3 may also be a promising target for the future treatment of AD. However, another study also found decreased neuronal m6A levels along with significantly reduced METTL3 expression in AD brains [48]. Interestingly, reduced neuronal m6A modifications in the hippocampus caused by METTL3 knockdown led to significant memory deficits, accompanied by extensive synaptic loss, neuronal death, and multiple AD-related cellular alterations [48]. Mechanistically, soluble Aβ oligomers caused a reduction in METTL3, whereas METTL3 overexpression rescued Aβ-induced synaptic damage, cognitive impairment, and postsynaptic density protein 95 (PSD95) loss in vitro [48]. Consistently, the upregulated METTL3 also actively clears the phosphorylated tau (p-Tau) to ameliorate AD [49]. For example, Tang et al. reported that lysine demethylase 1A (KDM1A) overexpression increased the expression of METTL3 in AD, which then stabilized STUB1 mRNA through the m6A-IGF2BP1-dependent mechanism to enhance STUB1 expression, thereby enhancing autophagic p-Tau clearance in Aβ1-42-treated cells [49]. Collectively, this study demonstrates that the novel KDM1A-METTL3/IGF2BP1-STUB1 pathway enhances autophagy-mediated clearance of p-Tau accumulation and thus ameliorates AD, which supports METTL3 as a potential therapeutic target for AD (Figure 2).

In summary, METTL3 controls the protein levels of key genes involved in AD-related pathways, suggesting a complex regulatory pattern of m6A signaling in AD-related pathogenesis that may be related to sample differences and different points of study.

### 4.4. METTL3 with Brain Tumors

GBM is the most common type of malignant brain tumor in adults [50]. Cancer cells in GBM tumors multiply rapidly and can spread into other areas of the brain but rarely metastasize to other parts of the body [50]. Glial cells, which are vital to nerve cell function, are the progenitors of GBM [51]. The pathogenesis of GBM is diverse, and treatments are not fully effective at present. An increasing numbers of studies have indicated a strong association of METTL3 with the occurrence and development of GBM. 

Studies have indicated the involvement of METTL3 in GBM via different mechanisms. For example, an in vitro study demonstrated that METTL3 can activate the Notch pathway and facilitate glioma development by regulating the pathway targets NOTCH3, delta-like protein 3(DLL3), and HES1 [52]. Consistent with this, Ji et al. reported that the expression of METTL3 was significantly lower in glioma tumor tissues than in adjacent normal tissues, and this downregulation mediated GBM occurrence by altering the PI3K/Akt pathway [53]. Other studies have indicated that METTL3 is downregulated in glioma tissues and regulates U251 cell proliferation and apoptosis by targeting COL4A1 and HSP9, potential therapeutic targets of glioma [54,55]. Indeed, there are already studies that provide insight into targeting METTL3 for GBM treatment. For example, in the blood–tumor barrier (BTB) model constructed by glioma microvascular endothelial cells (GECs), Zhang et al. reported that upregulation of METTL3 and IGF2BP3 increased the stability of CPEB2 mRNA through its m6A methylation [56], while CPEB2, in turn, binds to and increases the stability of the SRSF5 mRNA, promoting the inclusion of ETS1 exons [56]. Finally, ETS1 promotes transcriptional expression of ZO-1, occludin, and claudin-5, thereby raising BTB permeability, which is essential for GBM therapy [56]. Collectively, these data demonstrate that up-regulated METTL3 is therapeutic for GBM via the METTL3/IGF2BP3-CPEB2-SRSF5-ETS1 axis to raise BTB permeability. Moreover, Shi et al. reported that the METTL3 knockdown increased the self-renew, proliferation, and temozolomide (TMZ) half-maximal inhibitory concentration of glioma stem cells (GSCs) via reducing METTL3-mediated m6A modification of DNA repair gene (MGMT and APNG) mRNAs that subsequently inhibited TMZ sensitivity of GSCs [57]. Consistent with this, Lv et al. supported a GBM therapeutic role by targeting METTL3, which reported that upregulation of METTL3 expression by platelet-derived growth factor receptor (PDGFR) signaling promoted mitophagy regulator OPTN (optineurin) mRNA degradation to suppress tumor [58] (Table 1). 

The METTL3 expression level is also a potential prognostic indicator for patients with GBM. Tao et al. demonstrated that downregulation of METTL3 enhanced the invasive properties of GBM by regulating the expression of cadherin 1(CDH1), cadherin 2(CDH2), matrix metallopeptidase 2(MMP2), and fibronectin 1(FN1) [59]. They indicated that patients with relatively high expression levels of METTL3 had prolonged disease-free survival. However, it has been reported that fear stress may induce upregulation of METTL3 to promote tumor progression through inhibition of ferroptosis, which functions by enhancing the stability of FSP1 mRNA [60]. In addition, another study also indicated that METTL3 expression is positively associated with higher malignant grades and poorer prognoses in isocitrate dehydrogenase-wildtype gliomas but not isocitrate dehydrogenase-mutant gliomas, which further suggests that METTL3 can promote the malignant progression of gliomas [61]. Another study indicated that YTHDF2 expression was positively associated with higher malignant grade, a molecular subtype of glioma, and poorer prognosis, and accelerated UBXN1 mRNA degradation via METTL3-mediated m6A [61]; this in turn promoted NF-κB activation [62]. These findings reveal a complex regulation pattern of METTL3 in the survival of patients with GBM and suggest that METTL3 is a therapeutic target for GBM and a prognostic marker of malignant grade (Table 1).

### 4.5. METTL3 with Brain Injuries

Brain injuries usually manifest as stroke or TBI, with complex pathogenesis mechanisms [63]. Various studies have indicated the involvement of METTL3 in brain injuries. For example, using the middle cerebral artery occlusion (MCAO) model in rats and an oxygen-glucose deprivation (OGD)/reperfusion model in primary cortical neurons and PC12 cells, Si et al. reported that the expression of METTL3, m6A, and miR-335 was significantly decreased during the reperfusion period [64]. Either overexpression or knockdown of METTL3 in OGD PC12 cells resulted in functional maturation of miR-335, stress granule (SG) formation, and increased apoptotic levels [64]; METTL3 interaction with the miR-335/Erf1 signaling pathway promoted SG formation. Therefore, the METTL3/miR-335/Erf1 axis might represent a targeted therapeutic strategy against acute ischemic stroke (AIS). Furthermore, in a mouse model, researchers found that METTL3 was downregulated after TBI, which was regulated by the mitogen-activated protein kinase (MAPK) signaling pathway that targeted SOX21 [65]. In a zebrafish spinal cord injury (SCI) model, researchers also found that the expression of METTL3 was increased in both astrocytes and NSCs, suggesting that METTL3 is important for spinal cord regeneration [66]. In addition, Ge et al. reported that METTL3 is up-regulated in reactive astrocytes following SCI, which is further stabilized by the USP1/UAF1 complex that specifically binds to and subsequently removes the K48-linked ubiquitin [67]. Mechanistically, METTL3 selectively methylated the 3ʹ-UTR region of the YAP1 transcript to maintain its stability in an IGF2BP2-dependent manner in reactive astrogliosis [67]. Collectively, these data suggest that USP1/UAF1 complex-mediated METTL3 upregulation contributes to SCI occurrence and development via the METTL3/IGF2BP2-YAP1 axis. Moreover, METTL3 triggers the occurrence and development of sciatic nerve injury (SNI) as well. He et al. reported that METTL3 is upregulated in a rat model of SNI, enhancing the abundance of m6A-modified LncRNA D26496 and acting synergistically with YTHDF2 to enhance the recognition of D26496 m6A sites to induce D26496 degradation, thereby participating in the progression of SNI [68] (Figure 2).

### 4.6. METTL3 with Other Brain Disorders

Apart from involvement in tumors, degenerative disorders, and brain injuries, METTL3 has also been reported to be involved in other brain disorders. Wang et al. found that the expression of METTL3 was reduced in the pathological tissues of cerebral arteriovenous malformations [69]. Knockdown of METTL3 significantly affected the angiogenesis of human endothelial cells by reducing the level of heterodimeric Notch E3 ubiquitin ligase and activating the Notch signaling pathway [69]. In postoperative cognitive dysfunction (POCD) mouse models that were constructed using sevoflurane, researchers found that METTL3 was downregulated in the mouse hippocampus by sevoflurane-mediated MAPK/ERK suppression, and its target genes brain-derived neurotrophic factor (BDNF), SOX2, and synapsin 1(SYN1) were highly enriched at their 5′-UTRs [70]. Additionally, in a rat model of typical neuropathic pain, researchers found that METTL3 was considerably downregulated [71]. Mechanistically, METTL3 accelerated miR-150 maturation by mediating the m6A methylation of pri-miR-150 at locus 498, cooperating with the “m6A reader” YTHDF2. Furthermore, miR-150 could directly target BDNF mRNA. Therefore, the METTL3/miR-150/BDNF pathway might be a promising therapeutic target for patients with neuropathic pain [71] (Figure 2).

## 5. Discussion

METTL3 is a key catalytic component for m6A RNA modification, catalyzing the conversion of adenosine to m6A via its active methyltransferase domain [72]. Apart from its prominent methyltransferase activity, METTL3 regulates the translation of target mRNAs independent of its catalytic subunit. METTL3 has the highest expression level in the brain, suggesting an important relationship with the nervous system [72]. Moreover, several studies have reported the participation of METTL3 as a dynamic epigenetic regulator involved in the regulation of neurophysiological and neuropathological events. Thus, mapping the roles and mechanisms of METTL3 in regulating nervous system events would be helpful for both the clinical diagnosis and treatment of neurological diseases.

In this review, we summarized studies on METTL3 in the nervous system, analyzed its biological functions in regulating gene expression, highlighted the multifaceted roles and mechanisms of METTL3 in neurophysiological and neuropathological processes, and finally mapped out a regulation network for METTL3. This network showed that METTL3 could regulate nerve cells’ biological processes such as reprogramming, proliferation, apoptosis, and differentiation. In these different cell processes, METTL3 had different expression patterns and diverse mechanisms. Moreover, after mapping the regulation patterns of METTL3 in neurological events, we found that low expression of METTL3 primarily inactivates neurological events such as neurogenesis, while exacerbating neuropathological events, such as worsening GBM. In addition, we found that the down-regulated levels of METTL3 played different roles in the nervous system. However, in most of the publications, its down regulation inhibited normal neurophysiological events and triggered neuropathological events. In addition, METTL3 was used as a prognostic biomarker in most of the original studies. Therefore, our review suggests that METTL3 could be used as a diagnostic biomarker and therapeutic target in the nervous system. Furthermore, we found that some studies showed an increase, and others a decrease in METTL3 expression in glioblastoma. This may be related to the different sample origins used for detection, as well as different targets or pathways for METTL3 regulation. Therefore, determining the double-edged roles of METTL3 in neurological events is necessary.

Furthermore, we find that the role of METTL3 in nerve cells varies from neuronal stem cells to neurons to the pro-inflammatory phenotype of microglia to astrocytes. However, to our knowledge and in our previously published review [73], there have been few studies on the roles of METTL3 in oligodendrocytes and the anti-inflammatory phenotype of microglia. As anti-inflammatory phenotype microglia possess similar functions to those of macrophages and studies have indicated the important roles of METTL3 in regulating the activation of macrophages [74], we predict that METTL3 will be relevant in regulating anti-inflammatory phenotype microglia activation. Additionally, we found that METTL3 widely participates in neurophysiology and neuropathological events, but to our knowledge, few studies have indicated its roles in social interaction and developing brain disorders such as white matter injury, attention deficit hyperactivity disorders, and schizophrenia. Therefore, further studies on the relationship of METTL3 with developing brain disorders are warranted. Furthermore, although a study has reported the downregulation of METTL3 in amyotrophic lateral sclerosis (ALS), which is a progressive neurodegenerative disease resulting in the death of upper and lower motor neurons, its underlying mechanisms have not been clarified yet and needs further clarification [75].

## 6. Conclusions

In summary, METTL3 has been widely studied in the nervous system and exerts diverse roles and mechanisms in different events. This review suggested that METTL3 may be a potential diagnostic biomarker and treatment target for neurological diseases. We also suggest future study directions, such as exploring the roles and mechanisms of METTL3 in microglia and developing brain disorders.

## Figures and Tables

**Table 1:** Roles and Mechanisms of METTL3 in Brain Tumors.

Detection Source	METTL3 Expression	METTL3 Functions	Pathway/ Targets	Reference
Glioma stem cells (GSCs)	lower	Increase GSCs proliferation	Activate Notch signaling pathway, Reduce SOX2 DLL3, NOTCH3, HES1	[16,52]
GBM cell lines (U87MG and U118MG)	lower	Increase U87MG and U118MG proliferation	Decrease NOTCH3, DLL3, HES1	[52]
Glioma tumor tissues Human glioma cell lines U87 and LN229	Lower	Enhance proliferation while inhibiting apoptosis of U87 and LN229, promote the migration and invasion of U87 and LN229	Inactivating PI3K/Akt pathway Increase p-Akt/mTOR	[53]
Human glioma cell lines U87 and U251	lower	Promote proliferative and metastatic capacities of glioma cells. Stimulates the malignant development of glioma	Increase COL4A1	[54]
Human GBM	higher	Suppress tumor, GBM treatment	IGF2BP3-CPEB2-SRSF5-ETS1 axis	[56]
Human GSCs	lower	Inhibit TMZ sensitivity of GSCs, promote tumor progression	Reduce MGMT and APNG	[57]
Human GBM	higher	Inhibit GBM tumor progression, GBM treatment	Increase OPTN	[58]
Human GBM	lower	Enhance the invasive properties of GBM	CDH1,CDH2,MMP2, FN1	[59]
Male BALB/c-nude mice Human glioma cell lines U251 cells	higher	Inhibit ferroptosis Promote glioma tumor progression in mice	Enhance FSP1	[60]
Isocitrate dehydrogenase -wildtype gliomas	higher	Indicator of higher malignant grade and poorer prognosis	Activate NF-κB pathway Decrease UBXN1	[61,62]

## Data Availability

Not applicable.
